# The TRiC/CCT Complex at the Crossroads of Metabolism and Hypoxia in GBM: Implications for IDH-Dependent Therapeutic Targeting

**DOI:** 10.3390/ijms27010373

**Published:** 2025-12-29

**Authors:** Giusi Alberti, Giuseppa D’Amico, Maria Antonella Augello, Francesco Cappello, Marta Anna Szychlinska, Celeste Caruso Bavisotto, Federica Scalia

**Affiliations:** 1 Department of Biomedicine, Neurosciences and Advanced Diagnostics (BiND), University of Palermo, 90127 Palermo, Italy; giusi.alberti@unipa.it (G.A.); giuseppa.damico01@unipa.it (G.D.); mariaantonella.augello@unipa.it (M.A.A.); francesco.cappello@unipa.it (F.C.); celeste.carusobavisotto@unipa.it (C.C.B.); 2 Department of Precision Medicine in Medical, Surgical, and Critical Care Areas (Me.Pre.C.C.), University of Palermo, 90127 Palermo, Italy; 3 Department of Medicine and Surgery, Kore University of Enna, 94100 Enna, Italy

**Keywords:** TRiC/CCT, α-ketoglutarate (αKG), prolyl hydroxylase domain (PHD), hypoxia-inducible factor (HIF), metabolism, glioblastoma, cancer stem cells, tumorigenic processes, therapy, GBM

## Abstract

Glioblastoma (GBM) is characterized by its unique molecular features, such as self-renewal and tumorigenicity of glioma stem cells that promote resistance, largely resulting in treatment failure. Among the molecular alterations significant to GBM biology and treatment, mutations in isocitrate dehydrogenase (IDH) have assumed particular relevance. IDH-mutant and IDH-wild-type tumors exhibit significantly different metabolic characteristics, clinical behavior, and therapeutic sensitivities, making IDH status a critical determinant in determining prognosis and treatment strategies for GBM. In the context of cancer, chaperones were shown to promote tumor progression by supporting malignant cells over healthy ones. While heat shock proteins (HSPs) have long been implicated in the molecular mechanisms of tumor phenotype progression, recent attention has turned to CCT (chaperonin containing TCP1), orchestrating proteostasis. The chaperonin CCT is being explored as a diagnostic and therapeutic target in many cancers, including GBM, owing to its involvement in key oncogenic signaling pathways such as Wnt, VEGF, EGFR, and PI3K/AKT/mTOR. However, its role in the GBM-tricarboxylic acid (TCA) cycle cascade is still not well understood. Therefore, the present review highlights the potential role of the CCT complex in regulating hypoxia-inducible factor (HIF) activation by modulating enzymes responsive to metabolites derived from glucose metabolism and the TCA cycle in a manner dependent on oxygen availability and IDH mutation status.

## 1. Introduction

Glioblastoma multiforme (GBM) is a highly aggressive form of cancer. The current standard of care for newly diagnosed GBM consists of a maximally safe surgical resection, followed by the administration of the chemotherapy drug temozolomide (TMZ) and daily radiotherapy, which allows for an improvement in median overall survival (OS) and total survival of approximately 2 years [1,2,3]. Although resection is a necessary and safe surgical step with increased survival, the risk of neurological damage is a warning that should not be underestimated. Generally, different treatment protocols are followed depending on the patient’s age and stage of disease [1,4]. Unfortunately, chemotherapy or biotherapy in addition to concomitant chemoradiation for newly diagnosed GBM has not shown an incremental survival benefit [1]. In 2009, bevacizumab was approved by the US FDA as an antiangiogenic drug for GBM patients. Although it does not bring great benefits in terms of survival, it is useful for reducing peritumoral edema. The survival advantages are improved when used in combination with other angiogenic drugs, such as carboplatin, irinotecan, etoposide, erlotinib, and cetuximab [1,2,5]. Nonetheless, treatments fail mainly due to the peculiar molecular characteristics of GBM, characterized by the presence of stem cells, i.e., glioma stem cells (GSCs), which possess self-renewal and tumorigenicity potential, making it resistant to the treatments [6]. 

Recurrent GBMs are associated with the worst prognosis, and there is no standard of care in this clinical situation [5]. Depending on the location and size of the tumor, a new surgical resection may be considered [1]. Patients who undergo reoperation are usually younger, with better Karnofsky Performance Status (KPS ≥ 70) and smaller tumor size [5]. Surgery can also be useful to confirm the diagnosis and to identify target mutations for clinical trials directed toward personalized therapy [1,7]. Reirradiation remains a palliative option, but an uncertain curative therapy [1,5]. Specifically, chemotherapy treatment with TMZ is considered in recurrent GBMs for patients who have had a response to the first treatment, while nitrosoureas are used for patients who have not responded to TMZ [1,5]. 

For these reasons, more studies on the genetic, epigenetic, and metabolic tumor characteristics of GBM are crucial to find new molecular therapeutic approaches. Currently, molecular characteristics that offer crucial diagnostic and prognostic information for targeted therapies have been added to the GBM classification, going beyond histopathology [8,9,10]. In the 2016 revision of the World Health Organization’s classification of CNS tumors, the determination of IDH mutation status was introduced, distinguishing GBM subgroups, namely IDH-wild-type, IDH-mutant, and NOS (not otherwise specified) [2], with three variants of IDH wild-type, i.e., giant cell GBM, gliosarcoma, and epithelial-like GBM (Ep-GBM) [11]. GBM patients with IDH1/IDH2 mutations have been shown to have a higher survival rate than those without such mutations, with a median survival of 24–31 months compared to 9–15 months for wild-type IDH [12]. Under the 2021 classification update, the term GBM is used only for wild-type IDH gliomas, the most aggressive form [13]. However, for the sake of consistency with the literature and to facilitate discussion of studies predating this classification change, in this review, we will also refer to IDH-mutant tumors as GBM. Nonetheless, these classifications, although valuable for the precise categorization of gliomas, remain insufficient for defining reliable treatment strategies, and the array of molecules implicated in various tumor conditions still requires extensive exploration. 

Molecular chaperones, including heat shock proteins (HSPs), are elements that typically protect cells; however, when dysregulated, they can contribute to diseases known as chaperonopathies, including neurochaperonopathies that affect the nervous system [14,15,16,17,18]. In cancer, chaperones may promote tumor progression by supporting malignant cells over healthy ones. 

While HSPs have long been implicated in the molecular mechanisms of tumor phenotype progression, recent attention has turned to CCT (chaperonin containing TCP1), also known as TRiC (TCP-1 Ring Complex) [19,20]. The CCT chaperonin is emerging as a key molecule linked to uncontrolled proliferation, invasion, migration, and tumorigenesis [21,22] and its subunits are reported to play a key role in many tumors, such as neuroblastoma [23], lung adenocarcinoma [24], hepatocellular carcinoma [25], colon rectal cancer [26], breast cancer [27], and GBM [16]. Higher expression levels of CCT have been associated with tumorigenesis [19]. 

Together with pathogenic molecular pathways, metabolic features of GBM also play a crucial role in terms of tumor growth, invasion, angiogenesis, and the emergence of chemotherapy and radiation resistance. While the involvement of the CCT complex in the main GBM pathways, such as Wnt, TGF-β, VEGF, EGFR, CDKN2A, NF-κB, and the PI3K/AKT/mTOR, is under continuous observation and investigation by researchers [28,29,30], its role in the cascade belonging to GBM metabolic processes is less explored.

This review presents an overview of the CCT complex’s function in the modulation of hypoxia-inducible factor (HIF) activation by regulating enzymes sensitive to metabolic intermediates from glucose metabolism and the tricarboxylic acid (TCA) cycle. Its function under both normoxic (physiological) and hypoxic (pathological, GBM-related) conditions in neuronal cells, with reference to IDH–wild-type and IDH–mutant glioblastoma, is discussed. We suggest that the intricate role of the CCT complex, including its single subunits, in glucose metabolism and HIF regulation should be deeply investigated since it might be significantly influenced by the tumor’s genetic, epigenetic, and hypoxic conditions. In the final section of the manuscript, we discuss potential therapeutic strategies targeting the CCT chaperonin complex, with particular emphasis on its exploitation within combinatorial and multi-target therapeutic approaches. Understanding the underlying related factors could be useful in elaborating and specifying the patient’s tumor type, allowing clinical care to advance toward personalized, targeted treatment. 

## 2. TCA Cycle as a Target Therapy for Glioblastoma

The tricarboxylic acid (TCA) cycle is fundamental to cellular metabolism, as it underlies energy production, biosynthesis, and signaling processes. In GBM, this pathway is frequently dysregulated to sustain tumor growth and survival, thereby positioning TCA cycle enzymes as promising therapeutic targets. Although GBM cells predominantly use aerobic glycolysis, a metabolic adaptation known as the “Warburg effect”, they still exploit Krebs cycle intermediates to support anabolic demands and tumor progression. Consequently, several pharmacological agents, including Gboxin and metformin, have been developed to interfere with the Krebs cycle function and oxidative phosphorylation to interfere with energy homeostasis and mitigate tumor growth [31]. 

Interestingly, through analysis of GBM tissues, two subtypes can be distinguished: glycolytic-dominant, based on energy production from glycolysis, and mitochondrial-dominant, also characterized by a strong dependence on glutaminolysis, as glutamine is the second major energy substrate after glucose, and therefore this pathway is known to be upregulated in tumors [32,33]. In the latter subtype, inhibition of glutaminase (GLS), a key enzyme in glutaminolysis, has emerged as an effective strategy to suppress tumor proliferation [32]. A variety of GLS inhibitors have been reported in the literature to date: the inhibitor CB-839 was currently the most promising but also 6-Diazo-5-oxo-L-norleucine (DON), (unfortunately characterized by gastrointestinal toxicity) and its derivate methyl-POM-DON-isopropyl-ester, which has been shown to be effective in murine models together with other compounds, such as derivatives of 2-sulfonyl pyrimidine and methanodibenzo[b,f][1,5]dioxocin, that have demonstrated antitumor efficacy [34]. 

Other studies have shown that therapeutic modulation of multiple metabolic pathways, including the TCA cycle, can help to disrupt the tumor cell supply chain. Specifically, several metabolic inhibitors have been evaluated, including epicocconone (EPIC), an inhibitor of succinate dehydrogenase A (SDHA) that impairs the TCA cycle and mitochondrial respiration; arachidonic acid (AA), a cytosolic phospholipase A_2_ (cPLA_2_) inhibitor that reduces the release of free fatty acids; and 2-deoxy-D-glucose (2-DG), a glucose analog that blocks glycolysis by inhibiting hexokinase activity. It was seen that their simultaneous use dramatically reduced ATP production (up to 95%), halting the cycle and suppressing tumor proliferation [35]. Another potential strategy involves the inhibition of pyruvate dehydrogenase alpha (PDHA), a key enzyme in pyruvate oxidation. Compounds such as CPI-613 (devimistat), a PDHA inhibitor, have shown promising anticancer activity and are currently being evaluated in phase III clinical trials [36]. Additional therapeutic strategies targeting TCA cycle enzymes used 2-fluorocitrate (2-FC), which, alone or in combination with pro-oxidant agents, inhibits aconitase, helping to counteract tumor proliferation. Furthermore, metabolites such as oxaloacetate or citrate, combined with TMZ or 3-bromopyruvate (3-BP), reduce cell viability in vitro. The pharmacological inhibitors ivosidenib and vorasideni target IDH mutations, while modulation of succinate via 2-deoxy-D-glucose has been shown to improve mitochondrial metabolism and cell differentiation [37]. 

Finally, dimethyl fumarate (DMF), which has antitumor and neuroprotective effects, has also been reported to be effective in combination with temozolomide and radiotherapy [37]. 

Concerning these existing molecules that target the TCA cycle, the literature does not indicate any action on the CCT complex; nevertheless, their potential activity on the complex cannot be disregarded, and the CCT/PHD or CCT/VHL complex might be viewed as a biomarker for novel molecules by reprogramming metabolism in GBM. In Table 1, we summarize the current treatments and strategies for GBM, including those targeting the TCA cycle.

## 3. The CCT Complex in Tumors

The CCT complex is a type II chaperonin that assists in the folding of approximately 10% of cytosolic proteins [20]. Present in all eukaryotic cells, it is composed of nine subunits, labelled 1 to 8 or α to θ, including two forms of subunit 6 [20]. These subunits assemble into a double-ring barrel structure containing a central cavity where polypeptide folding takes place [21]. Each CCT subunit consists of an equatorial domain featuring the ATP-binding site, an apical domain responsible for substrate binding, and an intermediate domain that acts as a bridge between the equatorial and apical domains [38,39]. The exact number and identity of CCT client proteins are still debated, and many remain to be identified. Actin and tubulin proteins are considered the primary folding substrates of CCT complex [21]; however, the function of the CCT oligomer also extends to the modulation of the assembly of the complex between the Von Hippel-Lindau tumor suppressor protein (pVHL) and elongin BC, in cooperation with HSP70 [18,21,40,41]. Furthermore, CCT also binds to the actin filament that covers and separates the gelsolin protein, which appears not to be folded by the CCT, which could instead have a role in modulating its activity [21]. It is also reported that CCT also folds the respective regulators of actin and tubulin, namely Plp2p and Plp1p, as well as a series of proteins containing the WD40 domain, involved in fundamental biological processes such as transcription, chromatin modification, and secretion [38]. The anaphase-promoting complex (APC/C) and its activators, Cdh1 and Cdc20, are also obligate substrates, as both require CCTs for folding and functional activation [19,38,42]. CCT has been shown to mediate the release of Cdc20 from the mitotic checkpoint system (MCC), thereby promoting MCC disassembly and the onset of anaphase [42]. 

The CCT chaperonin is therefore emerging as a key molecule in cell division, due precisely to its essential role in the folding of several proteins involved in the process, but also of Plk1, p27, and PP2a, suggesting it could have a crucial role in uncontrolled cell proliferation [43]. CCT also folds wild-type p53, a frequently mutated tumor suppressor, and its depletion leads to the accumulation of misfolded p53 [19,21,44]. It also binds Stat3 (signal transducer and activator of transcription 3), an oncogenic transcription factor that leads to tumor formation and neoplasms [19,21]. 

Actin polymerization and assembly are fundamental for cell motility, but also for tumor migration and invasion mechanisms, and are controlled by a series of proteins; two of these are known to be interaction partners of CCT: gelsolin, already mentioned, and PAK4, which activates p21, a cell cycle regulatory protein [21,22].

All its actions, functions, and interactions are strongly correlated with the cancer progression process; in fact, higher CCT expression levels have been associated with tumorigenesis [19]. 

## 4. The CCT Complex in GBM

Studies have shown increased concentrations of CCT subunits in extracellular vesicles (EVs) obtained from tissues and fluids derived from patients suffering from various CNS disorders, including GBM tumors [45,46]. In particular, EVs obtained from CUSA (cavitron ultrasonic surgical aspirator) samples during GBM surgeries showed higher levels of all eight CCT subunits compared with those from low-grade gliomas, with CCT1, CCT2, CCT6A, and CCT7 being the most prominently upregulated [34]. Moreover, analyses of gene expression and DNA copy number showed that CCT2, CCT3, CCT5, CCT6A, and CCT7 are upregulated in GBM compared with normal brain tissue, while CCT2, CCT3, CCT5, CCT6A, and CCT8 display higher copy numbers than in low-grade gliomas [29]. The CCT6A gene, located at the 7p11.2 locus alongside EGFR, shows co-expression and co-amplification, suggesting its potential as a GBM biomarker through elevated levels in EVs [29]. Likewise, CCT2 has been proposed as a marker for circulating tumor cells, emphasizing the CCT complex’s diagnostic and therapeutic relevance in cancer [23]. Silencing of the CCT6A subunit through siRNA has been shown to impair GBM cell migration and invasion, as well as to disrupt the epithelial–mesenchymal transition (EMT) process [47]. Similarly, the CCT8 subunit exhibits elevated expression in gliomas, and its knockdown results in reduced cell proliferation, angiogenesis, migration, and invasion [48]. Furthermore, the CCT complex has been reported to interact with the oncogene KLHDC8A, enhancing cilia formation and activating the Hedgehog signaling pathway in GBM stem cells [49]. Collectively, these findings highlight the crucial role of CCT subunits in glioma progression and underscore their potential as therapeutic targets in GBM.

## 5. Role of the CCT Complex in the Metabolic Reprogramming of Healthy and GBM Cells 

The following sections explore the role of the CCT complex in metabolic reprogramming under different contexts. We will examine its functions in healthy nervous tissue as well as in GBM, considering both IDH wild-type and IDH-mutant conditions. The discussion aims to highlight how CCT-mediated protein folding may intersect metabolic pathways, potentially influencing tumor progression and cellular homeostasis.

### 5.1. Metabolic Programming in Healthy Normoxic Conditions

**Figure 1:**
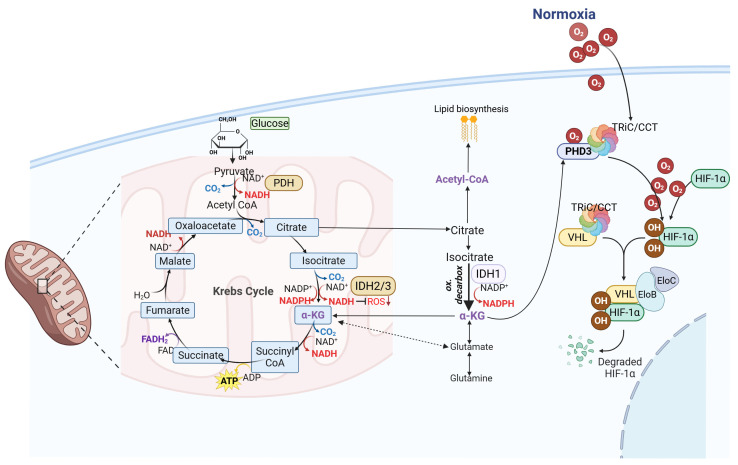
The role of wild-type IDH1 in normal (quiescent) human cells and its functional link to TRiC/CCT. Under normoxic conditions, glucose is metabolized to pyruvate through glycolysis. Pyruvate enters mitochondria, where it is converted by pyruvate dehydrogenase (PDH) into acetyl-CoA, fueling the tricarboxylic acid cycle (Krebs Cycle) and oxidative phosphorylation for ATP production. In this context, cytosolic wild-type IDH1 catalyzes the NADP⁺-dependent oxidative decarboxylation of isocitrate to α-ketoglutarate (α-KG), generating NADPH, which is essential for redox balance and lipid biosynthesis. IDH1 thereby contributes to the citrate–isocitrate–α-KG shuttle, linking cytosolic and mitochondrial metabolism. Beyond IDH1 activity, α-KG can also be derived from glutamine and glutamate through glutaminolysis (dashed arrow shows a pathway that is less strongly activated); this α-KG feeds the mitochondrial TCA cycle, supporting energy production and anaplerosis. Cytosolic α-KG serves as a cofactor for α-KG–dependent dioxygenases, such as prolyl hydroxylases (PHDs), which under normoxia hydroxylate HIF-1α, promoting its recognition by the pVHL-Elongin B and C (EloB and EloC) complex and subsequent proteasomal degradation. TRiC/CCT may regulate the activity of PHD3 and may support the folding and stabilization of pVHL protein. Thus, IDH1 and its metabolic products (NADPH, α-KG) may indirectly influence TRiC/CCT-dependent proteostasis under normoxic conditions. Created in BioRender. Caruso Bavisotto, C. (2025) https://BioRender.com/eshcd2.

One key property underlying cancer aggressiveness is the reprogramming of energy metabolism [50]. Glycolytic metabolism represents the primary energy pathway, both in the presence and absence of O_2_, thus ensuring cell survival and proliferation [51]. As reported above, this is known as the "Warburg effect" and is triggered by oncogenes and extended by metabolic and homeostatic alterations typical of tumors like GBM [52]. In normal (quiescent) cells, glycolytic metabolism is coupled to the mitochondrial TCA cycle and oxidative phosphorylation (OXPHOS). Specifically, pyruvate derived from glycolysis enters the mitochondrial matrix and is oxidized to acetyl coenzyme A (CoA) by the pyruvate dehydrogenase (PDH) complex [53] (Figure 1). 

A key metabolic enzyme for glucose metabolism and cellular respiration in the TCA cycle is IDH-1. In normal human cells, wild-type IDH-1 catalyzes the oxidative decarboxylation of isocitrate and simultaneously consumes a molecule of NADP+ to generate a molecule of α-ketoglutarate (α-KG), CO2 and NADPH (Figure 1). Notably, IDH-1 and IDH-2 enzymes utilize NADP+, while IDH-3 uses NAD+ as an electron acceptor to generate NADPH and NADH, respectively. Once produced, NADPH is involved in various cellular processes, such as defense against oxidative stress and fatty acid biosynthesis [54] (Figure 1). Furthermore, IDH-1 is also involved in lipid synthesis, amino acid utilization, and other processes [55]. The α-KG is an intermediate metabolite in an evolutionarily conserved pathway, i.e., the Krebs cycle [56], and is a central metabolic hub essential for the activity of α-KG-dependent dioxygenases (αKGD) localized in the cytoplasm (Figure 1). Specifically, the family of α-KG-dependent dioxygenases includes phylogenetically conserved enzymes, which include prolyl hydroxylase domain (PHD) proteins, whose activity is modulated by the intracellular concentration of iron and α-KG itself, and multiple demethylases involved in chromatin modifications [57,58]. PHDs exert an important role in regulating hypoxia-inducible factor (HIF) stability and activity, which is the main regulator of the response to low oxygen levels that typically occur in solid tumors [59]. Three isoforms of PHDs (termed PHD1, 2, and 3) are known, orthologues of the unique Egl-9 gene product, with different subcellular localizations: PHD1, expressed exclusively in the nucleus; PHD2, located in the cytosol; PHD3, expressed in almost equal proportions between the cytosol and the nucleus [60]. In addition, PHDs differ in their substrate specificity and inducibility of PHD genes, with both PHD2 and 3 mRNAs induced by hypoxic conditions, and PHD3 mRNA also induced by various other stimuli (such as p53 and nerve growth factor, NGF) [61]. Another difference lies in PHD mRNA tissue expression, with PHD2 mRNA the most abundant in most tissues, while PHD3 mRNA is expressed only in cardiac and neural tissue under non-stressed conditions [62]. Within the PHD’s family, PHD3 is the only dioxygenase with apoptotic properties in neural cells in oxygenated conditions [63]. Recently, a role as a tumor suppressor for PHD3 has been observed [64,65,66], in contrast to PHD1 and 2 [67,68]. In the presence of oxygen, PHDs post-translationally hydroxylate proline residues on the HIF-α subunit, regulating their degradation through ubiquitylation by the pVHL, with subsequent degradation by the 26S proteasome [66] (Figure 1). Nevertheless, HIF-α stability is also influenced by intracellular concentrations of α-KG, NOS, and other molecules [58]. 

The CCT chaperonins were found to bind PHD3 protein immediately at the end of mRNA translation, suggesting that PHD3 is a possible CCT substrate [69] (Figure 1). Specifically, the CCT complex establishes a stable interaction with PHD3 proteins through its subunits CCT1, CCT3, and CCT8 [69]. It was observed that the targeting of CCT complex by PHD3 appears to be specific for subcellular aggregates that PHD3 forms in an oxygen-dependent manner, as CCT may intervene by regulating the function/stability of PHD3 (Figure 1) [70]. However, it is consistent with both mutagenesis analysis of the CCT-pVHL binding determinants [71], as well as with the presence of a putative hydrophobic patch in the HL site of subunits CCT1 and CCT7, implicated in substrate binding [72]. Immunoprecipitation studies confirmed an interaction between PHD3 and the CCT chaperone, which is recognized as a component of protein aggregates, suggesting that CCT might control the activity of PHD3 [70]. Nonetheless, this hypothesis is an open question as it is unclear whether there is an interaction between the CCT-PHD3 and CCT-VHL complexes in vivo, which could impact HIF regulation. What is known is that 1) CCT-pVHL interaction in mammalian cells is necessary to mediate the formation of the VHL-elongin BC complex, able to regulate the E3 ubiquitin activation resulting in protein degradation [41], and 2) PHD3 tends to form aggresome-like structures, enriched in proteasomal components and various chaperones (including CCT) and ubiquitin, localized toward the perinuclear region, not surrounded by a vimentin cage, depending on the availability of O_2_ [70]. 

Therefore, based on literature data, it was hypothesized that these mechanisms might differ under hypoxic conditions, particularly between GBM with wild-type *vs*. mutant IDH.

### 5.2. GBM: Metabolic Reprogramming in the Wild-Type IDH Condition

**Figure 2:**
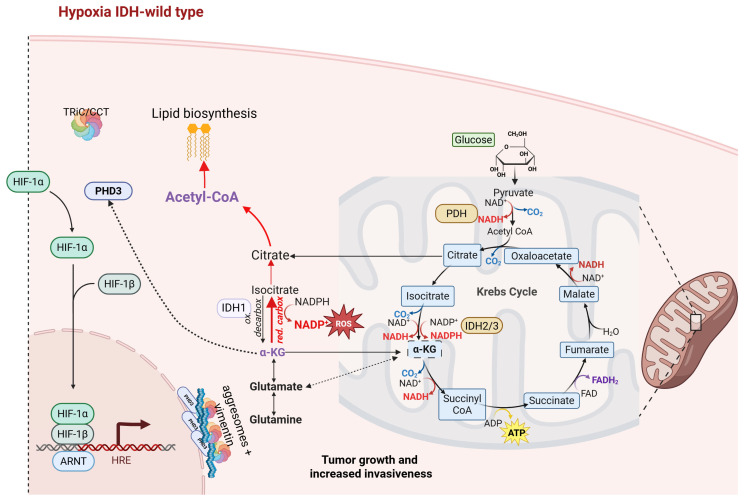
The role of wild-type IDH1 in GBM cells. In glioblastoma cells with wild-type IDH1, hypoxic conditions shift IDH1 activity from oxidative decarboxylation toward reductive carboxylation, using NADPH to convert α-ketoglutarate (α-KG) into isocitrate and citrate (red arrows). This metabolic rewiring supports lipid synthesis and tumor adaptation to hypoxia, while increased NADPH consumption may reduce redox buffering capacity. The α-KG produced or recycled through transamination of glutamate can also feed the mitochondrial TCA cycle, supporting anabolic growth and cell invasion (the dashed arrows show a pathway that is less strongly activated). Hypoxia also suppresses prolyl hydroxylase (PHD) activity, stabilizing HIF-α, which translocates to the nucleus and dimerizes with aryl hydrocarbon nuclear receptor translocator (ARNT), binding hypoxia response elements (HREs) to activate genes promoting angiogenesis, glycolysis, and survival. In parallel, hypoxic accumulation of PHD3 can lead to TRiC/CCT–PHD3–vimentin complex formation around the perinuclear region, contributing to cytoskeletal remodeling and enhanced tumor invasiveness through TRiC/CCT–pVHL chaperone interactions. Created in BioRender. Caruso Bavisotto, C. (2025) https://BioRender.com/eshcd2.

In recent years, the key role of wild-type IDH-1 in the progression of GBM has been increasingly underlined [73,74]. A typical property of GBM are the highly hypoxic areas where, instead, the carboxylation activity of wild-type IDH-1 is reductive and has been linked with tumor aggressiveness, invasion, as well as resistance to therapies [75]. In hypoxic conditions, IDH-1 activity shifts from its canonical oxidative decarboxylation reaction toward a glutamine-dependent reductive carboxylation pathway promoting lipid synthesis (Figure 2); unlike under normoxia, where lipids are synthesized from glucose [75]. 

Consequently, in this reverse flux, IDH-1 leads to the consumption of NADPH and production of NADP+. As a result, the cytosolic NADPH/NADP+ ratio can drop, weakening antioxidant defenses. In addition, lipid synthesis downstream of citrate also consumes NADPH, but this is a separate step, increasing the ROS susceptibility.

In hypoxic cells or decreased levels/function of α-KG condition, PHDs activity is compromised, resulting in the translocation of HIF-1α into the nucleus, where it regulates the transcription of target genes involved in cell growth, angiogenesis, metabolism, stem cell formation, and proliferation (Figure 2) [76,77,78]. As previously mentioned, PHD2 is the primary enzyme responsible for the hydroxylation of HIF-1α in normoxia, while PHD3 appears to intervene mainly in hypoxic conditions, preferentially on the hydroxylation of HIF-2α [58,59,60,61,62,63,64,65,66,67,68,69,70,71,72,73,74,75,76,77,78,79]. Compared to PHD2, PHD3 is the hydroxylase with the most robust response in low oxygen concentration [79]. Additionally, under limited oxygen conditions, evidence indicated that HIF-1/ 2α in turn transcriptionally regulates genes encoding PHD2 and 3 to promote accelerated HIF degradation in a negative feedback loop necessary to limit the hypoxic HIF response following re-oxygenation [80,81]. 

The CCT complex may indirectly influence the apoptosis process, modulating the folding/stability of the PHD3; since in GBM conditions, the hydroxylase activity of PHDs is reduced by the low oxygen concentration [82] and the decreased levels of α-KG, the CCT complex-dependent multiprotein complexes [83,84], including CCT-PHD3 [69], may not be formed, following inactivation of the hydroxylase function of PHD3 [70]. Collectively, these findings highlighted how hypoxic conditions could inhibit Krebs cycle-derived α-KGD activity through multiple mechanisms. However, this mechanistic scenario remains highly controversial, as both oxygen availability and the intracellular abundance of specific proteins critically determine the functional output of PHD3 and its interaction with chaperone systems. Gain and loss of function experiments of PHDs activity in a panel of GBM cell lines showed that PHD3, being a target gene of HIF-1α, remains operational even at low oxygen concentrations [80]. Furthermore, in GBM biopsies, PHD3 was found to be inducible by hypoxia and upregulated in hypoxic areas in vitro [80]. The hypoxic accumulation of PDH3 could be explained as the ability of GBM cells to rebalance the HIF activity threshold in response to the availability of surrounding oxygen [80]. However, under prolonged hypoxia, PDH3 remains mainly inactive, not inducing proteasomal aggregation [70,77]. A further layer of complexity arises from the involvement of CCT chaperonins. These complexes have been reported to exert both cytoprotective and cytotoxic roles, particularly in tumor settings where chaperone activity is compromised [21,85,86]. Under prolonged hypoxia typical of GBM, the accumulation of PDH3 together with the CCT chaperonins could lead to the formation of actual aggresomes capable of converging in the perinuclear region, thus contributing to the alteration of the organization of the cytoskeleton and leading to the formation of vimentin cages around them (Figure 2) [87]. These findings highlight that wild-type IDH-1, in coordination with the regulation of PHDs and the activity of the CCT complex, integrates metabolic processes, hypoxic response, and oxidative stress management, thereby contributing to the progression and survival of GBM cells. The hypoxic microenvironment characteristic of the tumor further modulates these interactions, suggesting that targeting IDH-1, PHDs, or CCT may represent a promising therapeutic strategy.

### 5.3. GBM: Metabolic Reprogramming in IDH Mutant Condition

The consequence of the IDH-1 somatic mutation in GBM profoundly alters its enzyme’s catalytic function. Rather than performing its normal physiological function, i.e., the oxidative decarboxylation of isocitrate (ICT) to α-ketoglutarate (α-KG) while producing NADPH, mutant IDH1 develops a neomorphic enzymatic property [88]

**Figure 3:**
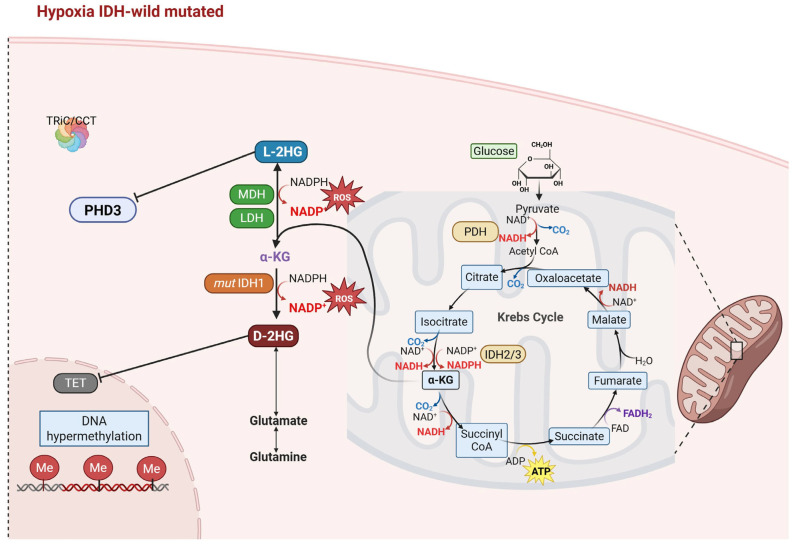
The role of mutant IDH-1 in GBM cells. Acquisition of the IDH-1 mutation results in major metabolic reprogramming in GBM cells. The neomorphic enzymatic activity of mutant IDH-1 reduces the Krebs cycle activity and the production of metabolites such as glutamine, glutamate, and branched-chain amino acids, which represent alternative sources of fuel for cellular metabolism. In the presence of an IDH-1 mutation, α-KG is reduced to the D-2HG oncometabolite, resulting in intracellular acidification and NADH/NAD^+^ concentration, while L-2HG is produced through promiscuous side-reaction of lactate dehydrogenase (LDH) and malate dehydrogenase (MDH). Once produced, D-2HG promotes a DNA hypermethylation phenotype by inhibiting α-KG–dependent TET dioxygenases and histone demethylases. Created in BioRender. Caruso Bavisotto, C. (2025) https://BioRender.com/eshcd2.

Specifically, patients carrying the IDH-1 mutation catalyze the NADPH-dependent reduction in α-KG to the metabolite (D)2-hydroxyglutarate (D2-HG), resulting in reduced levels of NADPH produced, and, therefore, modulating the regulation of HIF-1α [89,90] (Figure 3). 

The presence of D-2HG oncometabolite is strongly related to tumor cells harboring mutant IDH-1, as validated in both tissue metabolomics research and *in vivo* MR-spectroscopy evaluations in glioma patients [88]. At the cellular level, due to its structural similarity to αKG, D-2HG acts as an antagonist of α-KG, inhibiting all the enzymatic activities of αKG-dependent enzymes, including those of PHDs, and leading to DNA hypermethylation, increased mitochondrial oxidative metabolism, and promotion of enzymes involved in tumorigenesis, thus transforming the epigenetic environment and influencing hypoxia signaling pathways (Figure 3) [54,91,92]. This epigenetic reprogramming, known as the glioma CpG island methylator phenotype (G-CIMP), may influence the transcription of genes downstream of HIF-1α [93]

In contrast, reduced oxygen levels, typically of GBM, lead to an increase in intracellular acidification and NADH/NAD^+^ concentration, which in turn leads to the reduction in α-KG to L enantiomer (L-2HG) through promiscuous side-reaction of lactate dehydrogenase and malate dehydrogenase. Therefore, L-2HG is generated independently of IDH mutations. Compared to D-2HG, L-2HG appears to be a more potent inhibitor of the α-KGD [93,94]. Additionally, excessive D-2HG production causes a decrease in α-KG levels, inhibition of TET dioxygenases and histone demethylases, which could affect the levels of the HIF-1α subunit, and thus of the PHDs required to hydroxylate and promote HIF degradation via pVHL. Collectively, both isomers can influence dioxygenase activity; however, the D-2HG is a defining oncometabolite biomarker [95] influencing the nature of IDH-mutant cancers, the L-2HG is a more context-sensitive metabolite associated with the cellular redox state and the availability of oxygen in the microenvironment. Nonetheless, the molecular mechanism underlying HIF regulation in the context of IDH-1 mutation is not entirely clear. Thus, patients with mutant IDH-1 express higher levels of HIF-1α than wild-type tumors, consistent with the inhibitory effect of D-2HG on prolyl hydroxylases and the consequent stabilization of HIF-1α under hypoxic conditions [96,97,98,99,100]. Furthermore, the imbalance of proteostasis caused by an impaired chaperone system may compromise the proper folding of pVHL by the CCT complex and its assembly with partner proteins elongin B and elongin C [40,41,101], resulting in non-degradation of HIF-1α. This failure can result in the stabilization and accumulation of HIF-1α, which may remain predominantly non-nuclear or inoperative at the nuclear level, due to the DNA hypermethylation. Furthermore, while there is currently no evidence indicating that HIF-1α and CCT aggregates exist in GBM, it remains biologically reasonable, particularly in tumor-specific stress circumstances. The effects of the abundant non-nuclear HIF-1α are not yet established, but its involvement in other pathogenic signaling pathways, such as the Wnt pathway, has been demonstrated [102]. 

Overall, the acquisition of mutant IDH-1 results in substantial reprogramming of cellular metabolism, altering the TCA cycle and promoting the engagement of multiple compensatory and redundant mechanisms that support GBM growth. The role of CCT complex in this context, particularly in relation to the impaired function of PHD3, VHL and HIF-1α, warrants further investigation to elucidate its potential impact on HIF-dependent signaling and tumor biology.

## 6. Discussion

To date, GBM remains an aggressive tumor characterized by high recurrence rates and poor patient survival. In this context, the aim of the present review was to explore the altered metabolic pathways with a focus on the involvement of the CCT complex. 

In particular, the molecular pathway of the TCA cycle, its downstream pathways, and their possible alteration in patients with GBM have been examined in both IDH-1 wild-type and mutant forms and compared to the mechanisms found in healthy cells. The central role of the CCT complex at the intersection of protein homeostasis and cellular metabolism of the TCA cycle under both normoxic and hypoxic conditions is highlighted, where its involvement appears to differ significantly between normal cells, IDH-wildtype GBM, and IDH-mutant GBM, suggesting a multifaceted function that could be exploited for therapeutic purposes.

Briefly, under physiological conditions, CCT contributes to protein folding and metabolic homeostasis by supporting α-KG-dependent enzymatic activity and preventing abnormal hypoxia signaling. Additionally, the CCT complex regulates the establishment of the CCT-pVHL interaction, essential for stabilizing the pVHL protein, which can then bind to HIF and elongin BC complex, resulting in HIF degradation. Consequently, the existence of the CCT complex appears to be crucial for maintaining the equilibrium of interactions among key proteins associated with cancer progression and for their stabilization inside the cells.

In IDH-wildtype GBM, however, hypoxia alters the metabolic cascade, which is mainly balanced toward the oxidative decarboxylation of α-KG and a depletion of this molecule from the TCA cycle, which in turn is not able to provide sufficient activation of α-KG-dependent enzymes (i.e., PHDs enzymes), despite the presence and binding to CCT complex. The results are: (1) aggresomes (TRiC-PHD3) formation around the nuclear membrane, which trap vimentin protein; (2) compromised folding and stabilization of pVHL protein, leading to non-degradation of HIF and its translocation into the nucleus; (3) increased lipid biosynthesis; (4) high oxidative stress; (5) increased tumor aggressiveness. This shift from a cytoprotective role to a tumor-promoting one suggests that targeting CCT aggregation or restoring its regulatory interaction with pVHL may represent a new therapeutic strategy. In contrast, in IDH-mutant GBM, metabolic rewiring mediated by D-2HG production modifies CCT involvement, promoting PHD2 activity and partially restoring HIF regulation. In this context, the role of CCT is unknown; it may exert a protective function by maintaining redox balance and limiting tumor progression, or it may impair cell proteostasis. 

Furthermore, although increased expression of CCT subunits has been reported in several brain tumors, including astrocytomas and glioblastomas [29,34], their correlation with IDH mutation status has not yet been clearly established. Data derived mainly from astrocytoma patients suggest an inverse relationship, with higher CCT expression predominantly observed in IDH-wild-type tumors [103]; in contrast, studies on glioblastoma have not specifically linked CCT overexpression to IDH mutation status [48]. In addition, mutations in genes encoding CCT complex subunits are not commonly reported as recurrent somatic alterations in glioblastoma patients. Nevertheless, the significance of mutations in IDH1 and IDH2 proteoforms can differ based on the type of tumor and associated genetic changes [104]. Therefore, there is a clear need for the quantitative assessment of both wild-type and mutant IDH expression, together with potential alterations in the CCT complex, as this combined analysis may provide deeper insights into glioblastoma biology.

Overall, these findings indicate that CCT plays a context-dependent role in GBM and may represent a promising target for therapeutic modulation of tumor metabolism.

## 7. Future Directions: TRIC/CCT Therapeutic Implications in GBM

Although no clinically approved drugs directly targeting the TRiC/CCT chaperonin complex are currently available, multiple lines of evidence support the concept that TRiC/CCT represents a druggable vulnerability in cancer, particularly in tumors characterized by high proteotoxic and metabolic stress. Direct pharmacological inhibition of TRiC/CCT remains challenging due to its essential role in normal cellular homeostasis; however, several experimental strategies have been described. These include peptide-based approaches such as CT20p, a peptide derived from Bax that has been demonstrated to specifically bind to the CCTβ subunit and preferentially trigger apoptosis in cancer cells, providing proof-of-concept for targeted CCT intervention [23,105]. Additional studies have explored small molecules that interfere with the ATPase cycle or conformational dynamics of TRiC/CCT, although these compounds currently lack sufficient specificity and remain limited to preclinical in vitro studies [106]. In this scenario, tumors harboring IDH mutations may display enhanced dependency on TRiC/CCT due to altered NADPH levels, redox imbalance, and higher accumulation of misfolded proteins, suggesting a potential therapeutic window for TRiC/CCT-centered combinatorial strategies.

Considering that TCA-targeting treatments (such as CPI-613, metabolic inhibitors) can inhibit tumor growth, it is particularly attractive to broaden the therapeutic approach to include proteostasis/chaperone networks as additional targets. The use of multi-target therapies is not unprecedented in the GBM treatment: for instance, Yang and collegues showed that a triple therapy combining a TCA-cycle inhibitor (small molecule inhibitor “EPIC-0412”), a cytosolic phospholipase A2 inhibitor, and a hexokinase II (HK2)-inhibitor 2-DG, dramatically reduced ATP production (up to 95%), decreased cell proliferation, induced G_0_/G_1_ cell cycle arrest, and suppressed tumor growth *in vitro* and *in vivo* [35]. Furthermore, it has been observed that specific inhibitors of the endo-β-glucuronidase heparinase enzyme, which does not participate directly in classical metabolic pathways such as glycolysis or the TCA cycle, may profoundly influences cellular metabolism indirectly through its effects on autophagic flux and modulating key oncogenic signaling pathways such as PI3K/AKT/mTOR and HIF-1α, both of which function as central regulators of metabolic adaptation [107,108]. Since GBM relies on both metabolic rewiring and chaperone-mediated stress responses, it is intriguing to hypothesize that a multi-axis strategy, integrating TCA cycle interference with the modulation of chaperone and autophagy-dependent survival pathways, may target multiple metabolic GBM vulnerabilities, overwhelming the adaptive capacity that characterizes GBM.

The TRiC/CCT complex is a strong candidate due to its crucial role in folding essential cellular proteins; therefore, targeting the CCT complex may further overwhelm tumor proteostasis, and the HIF regulation could be indirectly disrupted, potentially altering the balance between adaptive hypoxia responses *vs*. metabolic collapse.

The mutational profile of IDH1 might significantly influence both metabolic susceptibility and chaperone reliance, indicating that personalized metabolic–proteostasis treatment tailored to IDH status could be an effective approach. It is reasonable to believe that the basal stress on redox balance and chaperone requirements may make IDH1-mutant GBMs more susceptible to additional metabolic disturbance and proteostasis disruption. 

Collectively, these findings position TRiC/CCT not as a classical drug target but as a critical node in cancer proteostasis networks that can be therapeutically exploited in a context-dependent manner.

## Figures and Tables

**Table 1:** Advantages and disadvantages of current treatments and strategies for GBM.

Treatment/Strategy	Advantages	Disadvantages
Surgical Resection	Improves overall survival; reduces tumor mass and intracranial pressure; provides tissue for histopathological diagnosis and molecular profiling	Risk of neurological damage; not always feasible (tumor location/size); surgical morbidity (infection, bleeding, postoperative edema)
Radiotherapy + Temozolomide (TMZ)	Current standard of care; increases median survival (~15–24 months)	Limited long-term efficacy; resistance often develops; radiation-related toxicity
Bevacizumab (anti-VEGF)	Reduces peritumoral edema; improves quality of life and symptom control	No significant survival benefit as monotherapy; potential for hypertension, thromboembolism, impaired wound healing
Bevacizumab + other angiogenic drugs	May slightly improve survival in combinations (e.g., with carboplatin, irinotecan)	Modest and non-reproducible benefits
TMZ rechallenge (for recurrence)	Potential benefit in patients with prior good response and MGMT-methylated tumors	Limited to select patients; often ineffective in resistant tumors
Nitrosoureas (e.g., Lomustine, Carmustine)	Alternative for TMZ-resistant or recurrent GBM	Limited efficacy; hematologic toxicity (myelosuppression) and cumulative side effects
Reirradiation	Provides palliative benefit and symptom control	Not curative; limited by cumulative radiation dose and toxicity
Reoperation for recurrence	Enables tumor reduction and updated molecular profiling	Surgical morbidity and recovery time
Molecular/Genetic Profiling (e.g., IDH, MGMT, EGFR)	Enables personalized therapies and better prognostic stratification	Limited availability of effective targeted therapies for most genetic alterations; requires specialized facilities and may increase cost
IDH Inhibitors (e.g., Ivosidenib, Vorasidenib)	Targeted treatment for IDH-mutant GBM; improved survival in selected patients	Applicable only to IDH-mutant cases (minority); resistance can develop over time
CCT Complex Targeting (under investigation)	Potential future biomarker; involved in multiple tumor pathways	No direct therapies are available yet; still under early investigation
Gboxin	Targets oxidative phosphorylation selectively in GBM mitochondria; promising preclinical cytotoxicity	Needs further clinical validation; long-term effects unknown
Metformin	Inhibits mitochondrial metabolism and AMPK pathway; widely available and low-cost	Limited efficacy as monotherapy
CB-839 (GLS inhibitor)	Blocks glutaminolysis in oxidative GBM subtypes; promising preclinical data	Clinical efficacy still under investigation; not effective in all subtypes
6-Diazo-5-oxo-L-norleucine (DON)	Broad glutamine pathway inhibition; potent antimetabolic effects in preclinical models	Severe gastrointestinal toxicity
methyl-POM-DON (DON derivative)	Improved safety and bioavailability and enhanced preclinical efficacy compared to DON	Still in early-stage research; no clinical data yet
Epicocconone (EPIC)	Inhibits succinate dehydrogenase (SDHA), disrupting the TCA cycle; potential metabolic vulnerability	Specificity and human safety profile still unknown
Arachidonic Acid (AA) inhibitors (e.g., cPLA_2_)	Disrupt fatty acid metabolism and inflammatory signaling	Potential off-target metabolic disruptions
2-Deoxy-D-glucose (2-DG)	Inhibits glycolysis; enhances other metabolic inhibitors	Non-specific cytotoxicity; toxicity at high doses
CPI-613 (Devimistat)	Inhibits pyruvate and α-ketoglutarate dehydrogenase	Efficacy and side effects in GBM still under investigation
2-Fluorocitrate (2-FC)	Inhibits aconitase in the TCA cycle; effective in vitro	High toxicity risk
Oxaloacetate/Citrate + TMZ or 3-BP	Potential synergy with standard chemotherapy; effective in vitro	In vivo efficacy not yet confirmed
Dimethyl Fumarate (DMF)	Antitumor and neuroprotective properties; synergistic with TMZ and radiotherapy in preclinical models	Requires GBM-specific clinical validation; long-term safety unclear

## Data Availability

No new data were created or analyzed in this study. Data sharing is not applicable to this article.

## Abbreviations

The following abbreviations are used in this manuscript:

AA	arachidonic acid
ARNT	aryl hydrocarbon nuclear receptor translocator
3-BP	3-bromopyruvate
CCT	chaperonin containing TCP1
CDKN2A	cyclin-dependent kinase inhibitor 2A
CoA	acetyl coenzyme A
cPLA_2_	a cytosolic phospholipase A_2_
CNS	Central Nervous System
CS	chaperone system
CUSA	cavitron ultrasonic surgical aspirator
2-DG	2-deoxy-D-glucose
DMF	dimethyl fumarate
DON	6-Diazo-5-oxo-L-norleucine
D2-HG	(D)2-hydroxyglutarate
EGFR	epidermal growth factor receptor
EMT	epithelial–mesenchymal transition
Ep-GBM	epithelial-like GBM
EPIC	epicocconone
EVs	extracellular vesicles
2-FC	2-fluorocitrate
GBM	Glioblastoma
GLS	glutaminase
GSCs	glioma stem cells
HIF	hypoxia-inducible factor
HSPs	Heat shock proteins
ICT	isocitrate
IDH	isocitrate dehydrogenase
KPS	Karnofsky performance status
MCC	mitotic checkpoint system
mTOR	mammalian target of rapamycin
NADH	nicotinamide adenine dinucleotide
NF-κB	nuclear factor kappa-light-chain
NOS	not otherwise specified
2-OG	2-oxoglutarate
OXPHOS	oxidative phosphorylation
PDHA	pyruvate dehydrogenase alpha
PHD	prolyl hydroxylase domain
PI3K	phosphatidylinositol 3-kinase
pVHL	Von Hippel Lindau tumor suppressor protein
SDHA	succinate dehydrogenase A
ROS	Reactive Oxygen Species
TCA	tricarboxylic acid
TGF-β	Transforming Growth Factor Beta
TRiC	Tailless complex polypeptide 1 ring complex
TMZ	temozolomide
US FDA	United States Food and Drug Administration
VEGF	aascular endothelial growth factor
Wint	Wingless
WHO	World Health Organization
αKG	α-ketoglutarate
αKGD	α-KG-dependent dioxygenases
